# Safety and feasibility of cerebrolysin in treatment of primary intracerebral hemorrhage (CLINCH)—a prospective, randomized, open-label, blinded endpoint pilot trial

**DOI:** 10.3389/fneur.2025.1602956

**Published:** 2025-10-01

**Authors:** Adam Kobayashi, Kinga Rutkowska, Katarzyna Gocyla-Dudar, Beata Chelstowska, Natalia Pozarowszczyk, Michal Karlinski

**Affiliations:** ^1^Institute of Medical Sciences, Faculty of Medicine – Collegium Medicum, Cardinal Wyszynski University, Warsaw, Poland; ^2^Institute of Psychiatry and Neurology, Warsaw, Poland

**Keywords:** cerebrolysin, stroke, intracerebral hemorrhage, neuroprotection, outcome, mortality

## Abstract

**Background:**

Intracerebral hemorrhage (ICH) accounts for 15% of strokes with high mortality and limited treatment options. Cerebrolysin, a neuropeptide preparation with multimodal neuroprotective properties, has shown promise in acute ischemic stroke but remains inadequately studied in ICH.

**Methods:**

CLINCH is an investigator led, academic driven multicenter, prospective, randomized, open-label, blinded endpoint (PROBE) phase IV pilot study evaluating cerebrolysin in primary lobar ICH. We will randomize 88 patients with lobar ICH (30–80 mL; GCS 8–15; National Institutes of Health Stroke Scale, NIHSS ≥8) within 6 h of onset in a 1:1 ratio to receive either intravenous cerebrolysin (50 mL daily for 14 days) plus standard care including intensive rehabilitation, or standard care alone. Randomization will be stratified by ICH volume (30–50 vs. 51–80 mL) and GCS (8–12 vs. 13–15). Primary endpoints include 90-day mortality (safety) and functional independence (modified Rankin Scale score, mRS 0–2) at 90 days (efficacy). Secondary endpoints include neurological improvement on NIHSS, ordinal mRS shift, Barthel Index, hematoma expansion, and serious adverse events. Blinded assessors will evaluate clinical outcomes, with central adjudication of neuroimaging.

**Discussion:**

This trial addresses critical limitations of previous ICH neuroprotection studies by focusing on lobar hemorrhages, implementing an ultra-early treatment window (≤6 h), and combining neuroprotection with intensive rehabilitation. The restrictive eligibility criteria may limit generalizability but enhance the likelihood of detecting treatment effects. If positive, results would support a larger confirmatory trial and inform the sample size.

## Introduction

Intracerebral hemorrhage (ICH) accounts for approximately 15% of all strokes and carries substantial morbidity and mortality. Treatment decisions depend on multiple factors, including the patient’s age, comorbidities, clinical presentation, and neuroimaging (1). Over the last three decades, clinical trials in acute care and prevention have revolutionized everyday clinical practice in a stroke unit. However, the research on neuroprotection and neuroplasticity remains inconclusive (2, 3).

Several agents tested for neuroprotective effects after ischemic stroke, including verapamil, statins, argatroban, and glibenclamide (4) yield mostly neutral results. However, no medicinal product has been proven effective in ICH, neither to prevent direct brain damage nor to improve the long-term functional outcome. Hemostatic treatments such as tranexamic acid or factor VIIa have failed to show efficacy. At the same time, the clinical benefit from non-vitamin K oral anticoagulants (NOAC) reversal therapy is still uncertain and limited to the OAC-related hemorrhages (5, 6). Currently, the only evidence-based pharmacological intervention that improves outcomes after ICH is a treatment bundle of intensive blood pressure lowering combined with body temperature and glycemia control (7–10). However, hypoglycemic events linked to tight glucose control may be associated with increased mortality (11). The ENRICH trial has shown the benefit of minimally invasive surgical evacuation of moderately large lobar hematomas (12), which the recently announced MIND trial has partly supported (13).

Cerebrolysin is a mixture of peptides and amino acids isolated from purified porcine brain tissue with demonstrated anti-inflammatory activity (14). Its multipeptide composition confers multimodal effects on Tumor Necrosis Factor alpha (TNF-α), the Vascular Endothelial Growth Factor (VEGF), the Insulin-like Growth Factor (IGF-1) or the brain-derived NTF (BDNF) (15). In animal models, cerebrolysin positively affected behavioral performance, GABAergic synaptic current, as well as adrenergic and muscarinic modulation (16). Preclinical studies demonstrate its ability to inhibit neuroinflammation and apoptosis (17–20).

The proposed mechanisms by which cerebrolysin may improve the outcome after ICH include: (i) reduction of perihematomal inflammation through inhibition of pro-inflammatory cytokines; (ii) attenuation of excitotoxicity and oxidative stress in neurons surrounding the hematoma; (iii) promotion of blood–brain barrier integrity, potentially reducing edema formation; (iv) enhancement of neuroplasticity the recovery phase.

Clinical development of cerebrolysin demonstrates that this medication is generally safe and well-tolerated. In moderately-to-severe cortical syndromes, it augments early intensive post-ischemic stroke rehabilitation, resulting in statistically and clinically significant functional improvement (21–27). According to the European Academy of Neurology and the European Federation of Neurorehabilitation Societies guidelines, there are two interventions recommended to support early motor rehabilitation after acute ischemic stroke: (i) intravenous cerebrolysin 30 mL/day for a minimum of 10 days and (ii) citalopram 20 mg/day (28).

Conflicting conclusions of metaanalyses are caused by differences in the treatment window that in the neutral analysis excluded positive CARS-1/CARS-2 trials and were performed before the recently published ESCAS trial (DOI 10.1007/s10072-017-3214-0) (29). However, more recent studies also prove its’ positive effect on the risk of hemorrhagic transformation and functional outcome in acute ischemic stroke treated with reperfusion therapies (30–34).

Besides, the European Stroke Organization and the European Academy of Neurology in their guideline published in 2021 highlighted that there are not enough randomized clinical trials to confirm effectiveness of any mono- or polytherapy in the prevention of post-stroke cognitive functions decline. It was noted that the lack of clear definition of cognitive function results in a lack of agreement on what tests should be performed to measure these functions. While acknowledging cerebrolysin’s potential positive effects in vascular dementia, these guidelines highlighted insufficient data confirming effectiveness in post-stroke cognitive impairment (35).

To date no approved medicine mitigates the neurological and functional effects of hemorrhagic stroke. There is limited innovation in the development of novel neuroprotective therapeutics for ICH. Results from ischemic stroke trials provide a good rationale for investigating cerebrolysin in a well-designed randomized trial for the hyperacute ICH. One single study on an animal model of hemorrhagic stroke showed that administration of cerebrolysin results in reduced mortality and improved neurological outcome (36).

The observational retrospective study from 2019 that evaluated cerebrolysin treatment in patients with a minimally conscious state (MCS) observed a statistically significant improvement in the level of consciousness regardless the type of stroke (ischemic, hemorrhagic) (37).

Two trials have previously assessed cerebrolysin’s efficacy and safety in ICH. The first one demonstrating no effect after a 10-day treatment course. From the current perspective, those results must be reassessed due to several methodological limitations and advances in stroke care standards. The trial was conducted at a single center and enrolled patients with basal ganglia hematomas in whom neuroprotective and neuroregenerative effects are inherently challenging to demonstrate. The outcome measures were non-standard compared to those used generally for reporting stroke outcomes. Long-term outcomes were not reported, and the intervention did not involve early intensive rehabilitation (important for benefit from the synergistic effect of cerebrolysin and rehabilitation) and was initiated in a 6- to 24-h time window (38). This could also have affected the neutral outcome, as hematoma growth occurs mainly within 6 h of symptom onset (39). Another recent study showed a positive effect of cerebrolysion on functional outcome measured on the modified Rankin Scale (mRS) and Barthel Index and also reduced 6-month mortality (40) dose used in these patients was 30 mL daily over 21 days. Time from onset to needle was predefined as 12 h, nevertheless the patients were treated beyond 13 h of onset and less than 40% experienced lobar ICH.

The present CLINCH trial addresses these limitations by: (i) focusing exclusively on lobar hemorrhages where neuroprotective effects may be more readily detectable; (ii) implementing a pragmatic but narrow 6-h treatment window to consume the potentially effect on hematoma expansion; (iii) utilizing standardized, internationally recognized outcome measures; (iv) employing a multimodal assessment approach combining clinical, functional, and imaging outcomes; and most importantly (v) incorporating early intensive rehabilitation to maximize potential recovery benefits.

In Poland, cerebrolysin is currently registered for the treatment of organic disorders, including mild dementia in 5–50 mL daily intravenous infusions for 10–20 days. The clinical experience with cerebrolysin in acute stroke (both ischemic and hemorrhagic) includes 20–50 mL infusions administered once daily in cycles between 10 and 21 days.

Based on nonclinical and clinical study results, participants may experience clinical benefits from study participation, though direct benefits cannot be guaranteed. The investigator at each study site (or delegate) will protect the wellbeing of subjects.

## Methods and analysis

### Study design and settings

This is an investigator-initiated, academic driven hospital-based, multicenter, prospective, randomized, open-label, blinded endpoint (PROBE), phase IV parallel-group trial planned in Polish stroke units with access to rehabilitation wards. It is aimed at the population of patients who are currently lacking effective alternative therapies (NCT06899464).

The Faculty of Medicine, Collegium Medicum, Cardinal Stefan Wyszynski University in Warsaw, Poland, serves as the study sponsor.

A complete list of participating sites is provided on the clinicaltrials.gov website.

The study objective is to evaluate if a 14-day cerebrolysin treatment initiated within 6 h of onset of a primary lobar hemorrhage in addition to standard of care that includes early intensive rehabilitation is safe and feasible, affects hematoma growth and improves functional outcome.

Enrollment in the study is expected to reach 88 subjects over a 24-month period, and study completion (including follow-up) is anticipated within 15 months. The end of the study is defined as the date of the last patient’s last visit (LPLV). The sample size has been calculated based on the recent only positive trial in which patients with any type of ICH (including those with predominant basal ganglia involvement) and smaller volume (up to 25 mL) had 59.4% 6-month survival vs. 27.8% survival in patients receiving best medical treatment. Under the assumption of (i) similar absolute difference of 30%, (ii) alpha 0.05, and (iii) beta 0.80, we calculated the sample size for *N* = 88 (42 patients in each arm plus 5% for potential lost to follow-up) (40).

The PROBE design was chosen to simulate real-world practice, minimize cost and facilitate patient recruitment. Trial allocation will be known to investigators and patients, while study endpoints will be assessed by the blinded independent adjudicators.

The study protocol follows the Recommendations of Interventional Trials (SPIRIT) guidelines^1^.

### Participants

The study participants will be patients with acute ICH in whom allocated treatment can be initiated within 6 h of stroke onset and in whom a clinically meaningful benefit from treatment is considered possible. The full eligibility criteria are listed in Table 1.

**Table 1:** Inclusion and exclusion criteria.

Inclusion criteria	Exclusion criteria
1. Age 18–80 years	1. Hemorrhage caused by head trauma
2. NIHSS ≥8 at randomization	2. Medical history or neuroimaging findings suggestive of ruptured aneurysm, arteriovenous malformation (AVM), vascular anomaly, Moyamoya disease, venous sinus thrombosis, mass or tumor, hemorrhagic transformation of an ischemic infarct
3. Time from stroke onset <6h*	3. Intraventricular extension of the hemorrhage visually involving >50% of either of the lateral ventricles
4. Pre-randomization head CT demonstrating an acute, primary lobar ICH	4. Thalamic and basal ganglia ICH
5. ICH volume 30–80 mL	5. Infratentorial parenchymal hemorrhage, including midbrain, pons or cerebellum
6. Glasgow Coma Score (GCS) 8–15	6. Current use of low molecular weight heparins in therapeutic dose
7. No history of prior stroke	7. Uncorrected coagulopathy or known clotting disorder
8. Pre-stroke independence (modified Rankin Score 0–2)	8. Platelet count < 75,000, International Normalized Ratio (INR) > 1.4 after correction
9. Ability to provide informed consent	9. End stage renal disease
	10. Patients with a mechanical heart valve
	11. End-stage liver disease
	12. Epilepsy with grand mal seizure
	13. History of drug or alcohol use or dependence that, in the opinion of the site investigator, would interfere with adherence to study requirements
	14. Positive urine or serum pregnancy test in female subjects without documented history of surgical sterilization or post-menopausal
	15. Known life-expectancy of less than 6 months
	16. No reasonable expectation of recovery, Do-Not-Resuscitate (DNR), or comfort measures only prior to randomization
	17. Participation in a concurrent interventional medical investigation or clinical trial
	18. Inability or unwillingness of subject or legal guardian/representative to give written informed consent
	19. Any condition that would represent a contraindication for cerebrolysin administration

*Cerebrolysin treatment can be initiated within 6 h after the onset of stroke. If the actual time of onset is unclear, the time the subject was last known to be well will be used instead.

Written informed consent will be obtained by the investigator from each subject or their legal guardian before enrollment. The informed consent form (ICF) includes consent for pseudonymized data use according to the European Union General Data Protection Regulation (GDPR) requirements.

All enrolled subjects will be assigned a unique identification number in ascending order. Subject numbers will be used in the case report form (CRF). A list identifying the subjects by subject number will be kept in the site files.

### Intervention

The selected cerebrolysin dosage of 50 mL daily for 14 consecutive days is based on several considerations: (i) efficacy data from previous ischemic stroke trials suggesting that higher doses (30–50 mL) may produce more robust effects than lower doses; (ii) safety profile established in previous clinical studies confirming tolerability of this dosing regimen; (iii) the need for a sufficient treatment duration to potentially impact both acute (hematoma expansion, perihematomal edema) and subacute (inflammation, apoptosis) pathophysiological processes after ICH, and to support intensive rehabilitation.

The 6-h treatment window was selected based on the temporal profile of hematoma expansion in primary ICH, balancing (i) practical feasibility of patient identification, consent, and randomization; (ii) opportunity to affect hematoma expansion, which is the key determinant of functional outcome; (iii) 4- to 6-h therapeutic window for traumatic brain injury; and (iv) 8-h time window in the cerebrolysin thrombectomy trials.

The marketed product cerebrolysin 215.2 mg/mL solution for injection and infusion will be used as the study drug. For details and preparation instructions, please refer to the cerebrolysin Summary of Product Characteristics and package leaflet approved by the President of the Office for Registration of Medicinal Products, Medical Devices and Biocidal Products in Poland.

The cerebrolysin solution should not be mixed with amino acid solutions in one infusion. Vitamins and cardiovascular drugs may be administered simultaneously; however, they should not be mixed with a cerebrolysin solution in one infusion bag.

All subjects will receive care in the stroke unit and will receive stroke treatment and diagnosis according to Polish and international guidelines.

There are six time points of assessment (Table 2).

**Table 2:** Time points of assessment.

Time range	Assessment point
t0	Stroke onset
Day 1	≤5,5 h after t0: Baseline assessment prior to enrollment and randomization
Day 1	≤ 6 h after t0: Randomization followed by first dose of cerebrolysin (dosing in treatment arm only)
Day 2	24 ± 4 h from randomization
Day 7	7 days ±12 h from randomization
Day 14	14 days ±1 day from randomization
Day 30	30 days ± 2 days from Day 1
Day 90	90 days ± 7 days from Day 1

At baseline, brain non-contrast computed tomography (NCCT) and computed tomography angiography (CTA) scans, demographics, medical history, physical examination, vital signs, clinical scores [NIHSS, premorbid mRS and Barthel index (BI)], and eligibility criteria review will be recorded. The ICF signature will be collected prior to any study-specific procedure. Procedures completed as part of the standard of care but prior to ICF signature may be used for eligibility verification and baseline assessments.

All subjects, regardless of arm assignment, will have laboratory tests collected, and stroke treatment and rehabilitation programs will be implemented at the investigators’ discretion. Throughout the study, concomitant medications and adverse events (AEs) will be collected. Results will be investigator-assessed using Common Terminology Criteria for Adverse Events (CTCAE) version 5.0 (published November 27th, 2017).

Eight-eight subjects will be enrolled at 1:1 ratio.

After randomization, 44 subjects will receive an intravenous infusion of 50 mL cerebrolysin mixed with 250 mL of saline over 30 min once daily from Day 1 to Day 14 (14 consecutive days of treatment) plus standard of care treatment at the discretion of the investigator (treatment arm). The first dose of cerebrolysin must be administered as soon as possible and within 6 h from ICH onset. Another 44 subjects will be subject to receive standard of care treatment alone at the discretion of the investigator (control arm).

All subjects will be scheduled for intensive motor and/or speech and language rehabilitation, as stated in Polish reimbursement guidelines (120 min Per patient in patients with BI≥75 and 180 min Per patient in those with BI<75 day 5 days a week; additionally, all patients undergo 60 min Rehabilitation on Saturdays; in aphasic patients, speech therapy is additionally applied 5 days a week for 30 min).

Cerebrolysin will be administered once daily from Day 1 to Day 14 or until the end of treatment due to death, intolerable adverse events, subject loss to follow-up, consent withdrawal or study termination. Patients who discontinue treatment and receive at least one dose of cerebrolysin will be asked to attend all follow-up visits. A subject is considered as lost to follow-up when all follow-up efforts have been unsuccessful.

All subjects will undergo neurological assessments conducted by experienced stroke neurologists designated by the investigator: National Institutes of Health Stroke Scale (NIHSS) on Day 1 (at baseline), Day 2, Day 7, Day 14, Day 30, and Day 90; modified Rankin Scale (mRS) and BI on Day 1 (premorbid), Day 30 and Day 90.

The baseline brain NCCT and CTA will be completed on Day 1 (baseline) and the follow-up CT scan on Day 2 (24 ± 4 h from randomization) and day 14 ± 1 right after treatment termination. This will allow to assess the evolution of swelling which also reflects the status of blood–brain barrier permeability. The magnetic resonance imaging (MRI) scan will be completed on Day 90 ± 7 (41).

If on-site neurological assessment is not possible, investigators should schedule a telephone visit and collect information remotely.

**Figure 1:**
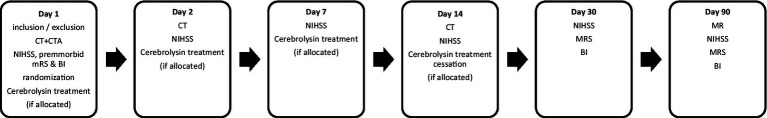
Trial assessments flowchart.

Please refer to Table 3 for study schedule details. The trial assessment flowchart is presented in Figure 1.

**Table 3:** Treatment schedule.

Treatment/procedure	Day 1 (screening)	Day 1 (baseline)	Day 2	Day 7	Day 14	Day 30	Day 90
Informed consent	x						
Eligibility assessment	x						
Demographics	x						
Medical history	x						
Physical examination*	x					x	x
ECOG score	x					x	x
Vital signs**	x					x	x
Pregnancy test, if applicable	x						
CT scan***	x		x		x		
MRI scan							x
mRS		x				x	x
NIHSS		x	x	x	x	x	x
Barthel index		x				x	x
Adverse events	x	x	x	x	x	x	
Concomitant medications	x	x	x	x	x	x	x
Randomization		x					
Cerebrolysin treatment (if allocated)		x	x	x	x		

*Physical examination: assessment of general appearance and review of organ systems (dermatologic, head, eyes, ears, nose, mouth/throat/neck, thyroid, lymph nodes, respiratory, cardiovascular, gastrointestinal, extremities, musculoskeletal, and neurologic systems). **Vital signs: pulse rate, respiratory rate, O2 saturation, body temperature, and blood pressure (systolic/diastolic, subjects should be supine/sitting for 5 min before assessment). ***Baseline brain NCCT and CTA scan on Day 1 (baseline) and follow-up CT scan on Day 2 (24 ± 4 h from randomization), and Day 14 (±1 day from randomization).

### Adverse events

At the time of study completion or early withdrawal, subjects will be asked directly about any AEs, concomitant medications, and concurrent procedures, which bias will be recorded in the source documentation and CRF. At the final study visit, subjects with ongoing AEs that are considered by the investigator to be study drug-related will be requested to continue to follow-up with the investigator until the AE resolves, stabilizes, or follow-up is no longer possible/necessary. Subjects will continue their prescribed medications and rehabilitation program following study completion. Any serious adverse event (SAE) that occurs while on study must be reported to the sponsor/designee for the study within 24 h after the site becomes aware of the event.

An SAE is defined as any untoward medical occurrence that at any tested drug dose: results in death, is life-threatening, requires hospitalization or prolongs existing hospitalization, results in persistent disability, is a congenital anomaly/birth defect or is medically important.

Medical and scientific judgment must be exercised in determining whether an AE is considered “medically important.”

Hospitalization is defined as (i) admission to a hospital/inpatient facility or (ii) staying at the hospital for treatment or observation for more than 24 h. Events leading to hospitalizations for the following reasons should not be reported as SAEs: (i) trial-related purposes not associated with any deterioration in condition, (ii) precautionary hospitalization for dosing of the study drug without any associated deterioration, (iii) social reasons in the absence of any deterioration in the patient’s general condition, (iv) elective surgery or other scheduled hospitalizations that were planned before the patient was enrolled for this trial.

Good Clinical Practice (GCP), European Medicines Agency pharmacovigilance, and national regulations for reporting adverse drug reactions (ADRs) and suspected unexpected serious adverse events (SUSAR) apply to both arms.

### Allocation

Participants will be stratified according to baseline ICH volume (30–50 mL vs. 51–80 mL) and Glasgow Coma Scale score (8–12 vs. 13–15) using randomised permuted blocks (block size 4) in a 1:1 ratio. Randomization will occur after baseline assessment using a computer-generated random allocation sequence. The treatment assignment will be provided to the site once a subject meets all eligibility criteria. The randomizing physician will be blinded to allocated treatment prior to randomization. Blocking details will be included in the Randomization Plan managed by the sponsor and will be unavailable to investigators enrolling participants and assigning interventions.

Subjects, healthcare providers, and data collectors will be aware of treatment allocation, while the independent outcome adjudicator will remain blinded.

### PROBE design

Clinical outcome assessments at 90 days will be performed by independent adjudicators (experienced stroke neurologists) who are not directly involved in the patient’s treatment and who are blinded to treatment assignment.

To ensure adequate blinding of outcome assessors, the following measures will be implemented: (i) assessors will have no access to treatment allocation information in medical records or study documentation; (ii) a site coordinator not involved in outcome assessment will accompany the patient and ensure no treatment-related discussions occur; and (iii) assessor blinding will be verified and documented after each assessment using a standardized questionnaire to evaluate potential unblinding.

A radiologist blinded to the treatment allocation will centrally adjudicate the baseline brain NCCT and CTA scan on Day 1 and the follow-up CT scan on Day 2 and a delayed follow-up CT scan on Day 14.

### Primary objectives

1. Safety outcome: mortality at day 90.
2. Efficacy outcome: regaining functional independence (mRS 0–2) at Day 90 following stroke onset.

### Secondary objectives

1. Neurological improvement from the deficit at baseline measured by NIHSS at Day 2, 7, 30, and 90.
2. Ordinal shift analysis of mRS at Day 90.
3. Improvement in activities of daily living from the status at baseline measured by BartheI Index on Day 30 and 90.
4. Avoiding hematoma growth from Day 1 to Day 2, defined as both absolute (>12.5 mL) and relative (>35%) increases in hematoma volume measured using semi-automated volumetric analysis on NCCT.
5. Absolute hematoma size shift.
6. SAE until day 30.

### Data collection, management, and monitoring

Study data will be collected as part of routine medical care. All data will be anonymized and reported using a CRF maintained by the sponsor. The investigator must ensure that data is entered into the CRF as soon as possible after the study visit.

Entries and corrections to the CRF data can be made by the investigator or delegated staff. Correction must include, at minimum, the original and corrected/changed data, identification of the person correcting/changing the data, and date and time of the correction/change.

Medical history and AEs will be coded using MedDRA Dictionary. Concomitant medication will be coded using the WHO Drug Dictionary.

In accordance with the ICH GCP principles, the sponsor will arrange study monitoring. Monitoring visits will be made at appropriate times to ensure that the trial is conducted and documented properly in compliance with the protocol, ICH GCP, and applicable local regulations. The sponsor designee (Clinical Research Associate, CRA) will review source documents to verify consistency with the CRF data (source data verification). The CRA will also provide information and support to the investigational sites. These activities will ensure data are attributable, legible, contemporaneous, original, and accurate.

Source data are defined as information in original records and certified copies of original records of clinical findings, observations, data, or other activities in a clinical study necessary for study reconstruction and evaluation.

### Statistical analysis

The study will be analyzed according to the intention-to-treat (ITT) principle. The primary analysis will include all randomized patients regardless of protocol adherence. A secondary per-protocol analysis will include only patients who received at least 80% of the planned cerebrolysin doses in the treatment arm and completed all required assessments.

If the patient’s treatment allocation was disclosed to the outcome adjudicator prior to the assessment, a substitute independent adjudicator will evaluate the patient. Safety analysis will apply to subjects who received at least one dose of cerebrolysin.

Details of the statistical analyses will be provided in the study’s statistical analysis plan (SAP), which will be finalized before enrolling the first patient. Any changes to the methods described in the plan will be described and justified in the final clinical study report.

Data will be summarized using descriptive statistics with 95% confidence intervals (CIs) where applicable. For continuous variables, the number of subjects, mean, standard deviation, median, minimum, and maximum values will be reported. Frequencies and percentages will be used to summarize categorical variables. Results will be presented as proportions or adjusted odds ratios with 95% confidence intervals and corresponding *p*-values. Analyses will be adjusted for the stratification factors (baseline ICH volume and GCS score). Missing data will be handled using multiple imputation methods for the primary and key secondary endpoints, with sensitivity analyses performed using alternative approaches (last observation carried forward, worst-case imputation) to assess the robustness of findings. A tipping-point analysis will be conducted for the primary endpoint to determine the impact of missing data on the final conclusions.

The following subgroup analyses are pre-specified according to age (≤65 vs. >65 years), baseline ICH volume (30–50 mL vs. 51–80 mL), the time from symptom onset to treatment (≤3 vs. >3–6 h), baseline GCS score (8–12 vs. 13–15).

All AEs and SAEs will be summarized, including the number of events, number of subjects, and percentage of subjects reporting these events, tabulated by preferred term from MedDRA Dictionary. Events will also be summarized by severity and by relationship to the study drug.

### Sample size

This is a pilot study with a target enrollment of 88 subjects at a 1:1 ratio. Such sample size has been considered sufficient to obtain stable means and standard deviations to inform the power calculation for the subsequent larger trial and the pace of recruitment. It has also the potential to confirm the large positive effect on mortality reported in a previous study.

### Limitations and strengths

This study has several limitations, particularly (i) the small sample size and (ii) only moderately long duration of follow-up. Potential for selection bias (lobar ICH, centers with rehabilitation access) and variability in standard of care across centers (BP targets, ICU protocols) carry a potential hazard. It has been designed to assess the early effect, safety, and feasibility of cerebrolysin in patients after ICH. If safety is confirmed and preliminary efficacy signals are observed, a subsequent trial with a larger sample size and longer observation period (at least 1 year) would be warranted, as ICH survivors may continue to improve over months after the stroke event.

The strengths of this trial include: (i) focus on a well-defined population (primary lobar ICH) that is likely to benefit from a neuroprotective and neuromodulating therapy; (ii) early intervention within the short and pragmatic therapeutic window (≤6 h); (iii) use of a PROBE design that maintains scientific rigor while ensuring recruitment feasibility; (iv) comprehensive assessment battery, (v) incorporation of early intensive rehabilitation to maximize potential recovery benefits; (vi) blinded central adjudication of both clinical and imaging outcomes.

The CLINCH trial embodies the concept of drug repurposing, investigating an established medication with a well-characterized safety profile in a new indication, potentially accelerating the translation of research findings to clinical practice. This is particularly valuable in a condition like ICH, where the development of completely novel compounds has proven challenging and where patients urgently need improved therapeutic options to reduce the substantial burden of disability associated with this devastating condition.

## Footnotes

^1^ https://spirit-statement.org
